# Urban heat island effect on cicada densities in metropolitan Seoul

**DOI:** 10.7717/peerj.4238

**Published:** 2018-01-12

**Authors:** Hoa Q. Nguyen, Desiree K. Andersen, Yuseob Kim, Yikweon Jang

**Affiliations:** 1 Interdisciplinary Program of EcoCreative, Ewha Womans University, Seoul, Republic of Korea; 2 Department of Life Sciences, Ewha Womans University, Seoul, Republic of Korea

**Keywords:** Urban heat island, *Cryptotympana atrata*, *Hyalessa fuscata*, Population dynamics, Cicada exuviae

## Abstract

**Background:**

Urban heat island (UHI) effect, the ubiquitous consequence of urbanization, is considered to play a major role in population expansion of numerous insects. *Cryptotympana atrata* and *Hyalessa fuscata* are the most abundant cicada species in the Korean Peninsula, where their population densities are higher in urban than in rural areas. We predicted a positive relationship between the UHI intensities and population densities of these two cicada species in metropolitan Seoul.

**Methods:**

To test this prediction, enumeration surveys of cicada exuviae densities were conducted in 36 localities located within and in the vicinity of metropolitan Seoul. Samples were collected in two consecutive periods from July to August 2015. The abundance of each species was estimated by two resource-weighted densities, one based on the total geographic area, and the other on the total number of trees. Multiple linear regression analyses were performed to identify factors critical for the prevalence of cicada species in the urban habitat.

**Results:**

*C. atrata* and *H. fuscata* were major constituents of cicada species composition collected across all localities. Minimum temperature and sampling period were significant factors contributing to the variation in densities of both species, whereas other environmental factors related to urbanization were not significant. More cicada exuviae were collected in the second rather than in the first samplings, which matched the phenological pattern of cicadas in metropolitan Seoul. Cicada population densities increased measurably with the increase in temperature. Age of residential complex also exhibited a significantly positive correlation to *H. fuscata* densities, but not to *C. atrata* densities.

**Discussion:**

Effects of temperature on cicada densities have been discerned from other environmental factors, as cicada densities increased measurably in tandem with elevated temperature. Several mechanisms may contribute to the abundance of cicadas in urban environments, such as higher fecundity of females, lower mortality rate of instars, decline in host plant quality, and local adaptation of organisms, but none of them were tested in the current study.

**Conclusions:**

In sum, results of the enumeration surveys of cicada exuviae support the hypothesis that the UHI effect underlies the population expansion of cicadas in metropolitan Seoul. Nevertheless, the underlying mechanisms for this remain untested.

## Introduction

Urbanization following rapid development and expansion in major cities usually incurs various microclimatic alterations, one of which is the urban heat island (UHI) effect (Oke, 1982; Wang et al., 2016). The UHI phenomenon occurs when the temperature of an urban ‘island’ becomes higher than the surrounding landscape (Oke, 1973; Rizwan, Dennis & Chunho, 2008). The phenomenon is primarily initiated by landscape modification from all types of land cover to dark impervious surface, and secondly due to anthropogenic heat release, which is related to human activities (Chen et al., 2006; Oke, 1982; Oke, 1995; Olfe & Lee, 1971; Wang et al., 2016). UHI is also modulated by vegetation cover that impedes such extreme conditions by cooling down summer heat and warming up winter nights (Myint et al., 2013; Wang et al., 2016). UHI varies seasonally, diurnally, and geographically; specifically, the intensities and frequency of occurrence of UHI depend on geographical location (Oke, 1982; Tzavali et al., 2015). In temperate mid-latitude cities, UHI is most pronounced in summer, whereas in high latitude areas, the most intense UHI is observed in winter (Oke, 1982). In metropolitan Seoul, variation of UHI intensities presents a temporal pattern where it is more prominent in winter than summer, in nighttime than daytime, and in weekdays than weekends (Kim & Baik, 2005). A similar pattern is also observed in Beijing (Yang, Ren & Liu, 2013). Consequently, thermal ranges experienced by organisms have been elevated in urban environments in recent decades (Brazel et al., 2000).

Urban warming caused by UHI (Memon, Leung & Liu, 2009) brings about deleterious ecological consequences, such as reduction in plant photosynthetic capability (Agrawal, 1998), limitation of water supplies to trees due to decrease in soil moisture (Jenerette et al., 2009), and decline in biological diversity (Willigalla & Fartmann, 2012). Those species who cannot adapt to such elevation in urban temperature are more vulnerable to extinction. Nevertheless, UHI is advantageous for those species able to exploit the higher temperatures available in urban areas for their development. For instance, growth rates of urban isolates of two fungi *Torulomyces lagena* and *Penicillium bilaii* are higher compared to rural isolates (McLean, Angilletta & Williams, 2005). In other cases, numerous scale insects are found to increase measurably from urban warming (Dale & Frank, 2014a; Meineke et al., 2013; Youngsteadt et al., 2014). A large amount of anthropogenic heat in cities may facilitate the abundance of herbivore pests by augmenting their fecundity and survival. For instance, the reproductive success of the gloomy scale insect *Melanaspis tenebricosa* females increased more than 50% with a 1.6 °C increase in ambient temperature (Dale & Frank, 2014b). Cicadas are likely to benefit from urban warming since their development and life history events require high thermal supplies (Moriyama & Numata, 2008). In western Japan, *Cryptotympana facialis*, a closely related species of *C. atrata*, has sharply increased in urbanized areas, owing to better thermal adaptation to warmer urban areas than to rural areas (Moriyama & Numata, 2008).

*Cryptotympana atrata* Fabricius (Tribe Cryptotympanini) and *Hyalessa fuscata* Distant (Tribe Sonatini) are two ubiquitous cicada species inhabiting the Korean peninsula (Lee, 2008). These two species are widely distributed in major cities, and their noisy calling songs in summer are a nuisance to city dwellers. In metropolitan Seoul, these two cicada species co-occur and are predominant among available cicada species. A previous enumeration survey of cicada exuviae revealed significantly higher densities of both species in urban and suburban areas compared to rural areas (Kim et al., 2014). Several hypotheses have been proposed to explain the high cicada density in an urban environment, such as host plant availability (Kim et al., 2014), predator avoidance strategy, habitat fragmentation (Takakura & Yamazaki, 2007), and urban soil compaction (Moriyama & Numata, 2015). Although these hypotheses have considered potential causes inducing higher cicada density in urban areas, to our knowledge none of them have properly explained the abundance of both *C. atrata* and *H. fuscata* in metropolitan Seoul.

Temperature is critical to life histories of cicadas, affecting muscle contraction (Fonseca & Revez, 2002), phenology (Moriyama & Numata, 2011; Sato & Sato, 2015), and distribution of the species (Toolson, 1998). Therefore, UHI as a result of urban warming may provoke population expansion of *C. atrata* and *H. fuscata* in metropolitan Seoul. In this study, we aimed to elucidate the effects of UHI phenomenon on population densities of those two cicada species. If UHI does, in fact, underlie the cicada population expansion, we expected: (1) temperatures of urban areas to be higher than those of the surroundings; (2) among environmental factors related to urbanization, temperature to be the most significant factor; and (3) cicada densities to increase measurably with the increase in temperature.

## Methodology

### Weather data

Firstly, we obtained meteorological data of 2014 from the Korea Meteorological Administration (http://www.weather.go.kr/weather/observation/aws_table_popup.jsp, accessed 21 May 2015). Daily minimum and maximum temperatures of 38 automatic weather stations within and in the vicinity of metropolitan Seoul were compiled from June 1 to August 31, 2014 (92 days in total). Among 38 weather stations, eight were determined as surrounding stations based on their geographic locations outside of the administrative boundary of metropolitan Seoul. This period was chosen because important life history events of these two cicadas, such as mating and oviposition, occur in summer. Moreover, although cicadas overwinter in diapause (Moriyama & Numata, 2008) and UHI is most intense in winter (Kim & Baik, 2005), the severity of winter weather conditions was not reported to influence hatching success of cicadas (Moriyama & Numata, 2009).

Averages of minimum and maximum temperatures in 92 days of each weather station were considered representative of minimum and maximum temperature of that station, respectively. Minimum and maximum temperatures of urban areas were 21.02 ±  0.99 °C (Mean ± SD) and 29.03 ± 0.66 °C, respectively; those of neighboring areas were 20.16 ± 0.72 °C and 28.58 °C, respectively. Independent-samples *t*-tests revealed significantly higher minimum temperature of urban stations compared to that of neighboring stations (*P* = 0.029) but no significant difference in maximum temperature between urban and neighboring stations (*P* = 0.093) (Supplemental Information 4). Therefore, minimum temperature was employed as an estimate of ambient temperature. Minimum temperature is also used in other UHI research since the difference in maximum temperatures between urban and suburban areas usually indicates a cooling rather than heating effect (Tzavali et al., 2015).

### Sampling localities

We randomly chose eight urban and four surrounding locations for sampling, each location corresponding to one weather station. Three localities in each location were further chosen as replicates. Localities were standardized as residential complexes where landscaping trees were usually found. Since all three localities within each location were examined thoroughly within one day in each sampling, the boundary of each locality was set within the geographic areas of about 10,000 m^2^. Besides, the distance between radii of any two localities was greater than 100 m, to avoid overlapping cicada ranges (Karban, 1981). Each locality encompasses two to six buildings within a residential area. Detailed information of each locality is provided in Table 1.

**Table 1:** Results of enumeration survey of cicada exuviae collected from 36 localities of 12 locations within and surrounding metropolitan Seoul. First sampling started from Jul 4 to Jul 18, second sampling from Jul 20 to Aug 3, 2015. Three localities of each location were sampled within one day in each sampling.

Location	Latitude	Longitude	Age of residential complex	#buildings	Total geographic area (m^2^)	Total #trees	1st sampling					2nd sampling				
							*Cryptotympana atrata*		*Hyalessa fuscata*		Other species	*Cryptotympana atrata*		*Hyalessa fuscata*		Other species
							Male	Female	Male	Female		Male	Female	Male	Female	
Yeongdeungpo	37.5340°N	126.9058°E	20	4	9,026	449	61	38	8	2	1	40	60	74	70	0
	37.5303°N	126.9080°E	13	5	9,407	293	24	19	5	1	0	8	5	24	20	0
	37.5295°N	126.9064°E	11	5	9,155	448	18	17	1	2	0	119	123	38	37	0
Gwangjin	37.5359°N	127.0714°E	26	5	9,530	398	68	28	50	17	0	107	157	188	160	0
	37.5334°N	127.0705°E	13	5	9,770	445	40	25	22	10	0	140	165	320	320	0
	37.5363°N	127.0744°E	27	5	9,826	236	7	1	24	10	0	26	20	145	120	0
Seocho	37.5021°N	127.0205°E	6	3	10,370	253	2	2	5	2	1	105	89	264	150	1
	37.4972°N	127.0228°E	36	3	10,501	274	15	5	174	130	8	142	118	1624	1339	43
	37.4934°N	127.0267°E	36	4	10,255	197	1	6	17	11	0	40	34	263	141	0
Gangbuk	37.4781°N	127.0178°E	15	3	9,474	252	1	0	5	0	0	0	0	56	31	0
	37.6430°N	127.0194°E	23	3	9,193	347	0	1	0	1	0	3	3	104	33	0
	37.642°N	127.0122°E	23	4	10,656	164	0	0	0	0	0	2	1	18	1	1
Dobong	37.6611°N	127.0339°E	27	3	9,030	288	0	0	0	0	0	0	0	13	4	0
	37.6636°N	127.0389°E	10	3	9,317	366	0	0	0	0	0	0	0	3	1	0
	37.6669°N	127.0475°E	13	4	9,585	506	0	0	0	0	0	0	0	9	4	0
Gwanak	37.4833°N	126.9105°E	12	4	10,130	417	8	5	0	0	0	38	36	14	3	0
	37.4764°N	126.9125°E	31	3	9,815	270	0	0	4	2	0	2	2	11	8	0
	37.4636°N	126.9311°E	10	3	7,596	347	0	0	0	0	0	1	1	24	2	0
Nowon	37.6308°N	127.0669°E	21	4	11,533	409	48	40	60	30	0	38	63	25	39	0
	37.6276°N	127.07°E	13	4	11,745	310	0	1	4	0	0	1	4	2	2	0
	37.6297°N	127.0719°E	21	3	11,298	338	0	0	1	1	0	0	2	3	0	0
Eunpyeong	37.6342°N	126.9203°E	6	4	9,667	349	0	0	0	0	0	0	0	1	0	0
	37.6337°N	126.9233°E	3	6	9,216	620	0	0	0	0	0	0	0	4	1	0
	37.6408°N	126.9194°E	5	3	9,706	320	0	0	0	0	0	0	1	0	0	0
Namyangju	37.6583°N	127.1455°E	8	4	10,782	229	0	0	0	0	0	0	0	0	0	0
	37.6558°N	127.1469°E	15	3	11,775	260	0	0	32	12	0	0	0	10	18	0
	37.64914°N	127.1439°E	21	3	11,210	187	1	0	12	4	0	0	0	14	17	0
Gwangmyeong	37.477°N	126.8736°E	29	4	10,434	328	5	2	4	0	0	12	14	88	53	0
	37.4807°N	126.8717°E	29	2	11,503	268	1	4	8	11	0	23	23	335	61	1
	37.4833°N	126.8703°E	30	5	10,200	326	3	0	6	6	0	36	27	183	88	0
Soha	37.4550°N	126.8853°E	6	4	9,829	384	41	33	0	2	0	42	34	2	0	0
	37.4515°N	126.8894°E	9	3	9,217	586	19	6	4	0	0	44	43	22	21	7
	37.4497°N	126.8919°E	5	4	10,054	292	0	0	0	0	0	0	0	0	1	0
Jookyo	37.6653°N	126.8344°E	10	3	10,330	355	0	0	0	0	0	0	0	2	1	0
	37.665°N	126.8375°E	29	4	9,740	143	4	4	0	0	0	4	2	1	0	1
	37.6632°N	126.8392°E	11	5	10,835	432	0	0	0	0	0	1	0	0	0	0

**Figure 1:**
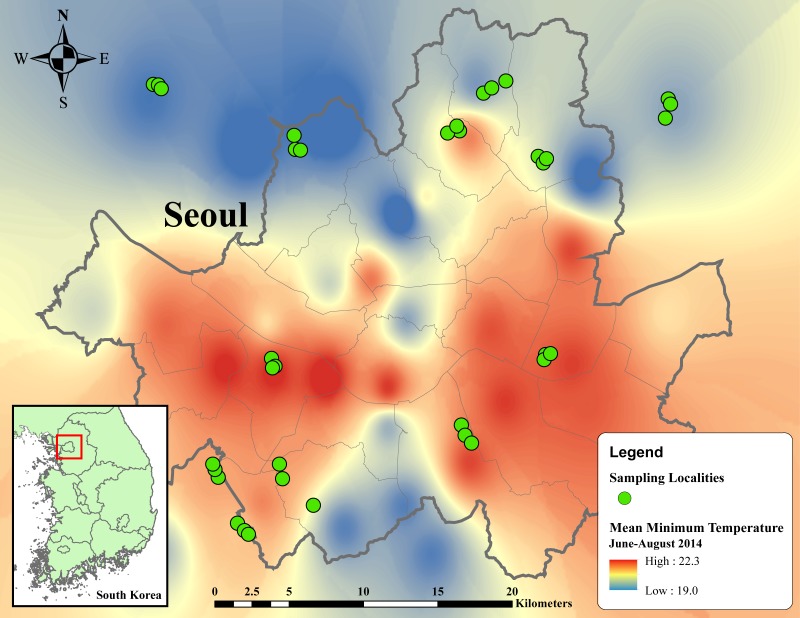
Thermal map of average minimum temperature from June to August 2014 in metropolitan Seoul interpolated from 38 weather stations using inverse distance weighted (IDW) interpolation in ArcMap 10.5. Temperature ranges from 19 to 22.3 °C, indicated by dark blue to red regions. Green circles designate sampling localities. Inset map in the below left corner illustrates the regional location of metropolitan Seoul in Republic of Korea.

We visited all localities in each location three times, with an interval between consecutive visits of at least 14 days. In the first visit, remaining exuviae of preceding broods were removed. The second and third visits respectively represented the first and second samplings of cicada emergence. In total, 36 localities of 12 locations were sampled twice from June 17 to August 3, 2015 (Fig. 1).

### Cicada exuvia collection

Since there is 1:1 matching between abundances of exuviae and adult cicadas, an enumeration survey of exuviae is a good predictor of cicada population density at a site (Moriyama & Numata, 2015; Sato & Sato, 2015; Takakura & Yamazaki, 2007). At the last developmental stage, the nymphs of the final cicada instars emerge from underground to molt. They shed their cast skins, or exuviae, on a nearby tree or on an artificial structure, then the adults fly away. These leftover materials can persist for a long time in the environment, even under exposure to variable environmental conditions. Accordingly, we relied on an enumeration survey of cicada exuviae to estimate population densities of the two target cicada species. Exuviae were collected from trees, underneath leaves, and on tree branches. Species identification of each exuvia was based on Lee, Oh & Jang (2012). Sexes were also determined from exuviae based on the morphology of the abdomen (Sato & Sato, 2015). Since there were no significant differences between the number of males and females in two sampling periods regardless of species (*P* > 0.05, Supplemental Information 5), the total number of exuviae was employed for the calculation of cicada population density.

We computed two types of resource-weighted densities as measurement of number of individuals living or using a resource (Lewontin & Levins, 1989): (1) area-weighted density, which is the number of exuviae divided by the total geographic area in a locality; and (2) tree-weighted density, which is the number of exuviae divided by the number of trees in a locality, regardless of the existence of exuviae. Area-weighted density indicates the number of cicadas living in a geographic unit, while tree-weighted density indicates the number of cicadas per available resource unit. Cicadas largely depend on trees during their lifetimes; they spend their nymphal stage underground, sucking fluid nutrition from tree roots; after emergence, imagoes continue relying on trees for food or as substrates for mating and oviposition. Consequently, trees can be considered as a resource for cicada population to exploit.

### Environmental variables

To determine environmental factors that may predict cicada density, we used the values of wetness, greenness, and imperviousness inferred from satellite data, as well as minimum temperature values previously collected from weather data. Wetness can be correlated to soil and vegetation moisture (Lobser & Cohen, 2007), while greenness represents the measure of “photosynthetically active vegetation”, and can, therefore, serve as a proxy for primary productivity (Lobser & Cohen, 2007). Imperviousness consists of building footprints as well as pavement and asphalt.

Greenness, wetness, and imperviousness were derived from corrected reflectance Landsat 8 OLI/TIRS layers 2–7 using tasseled cap transformations (Supplemental Information 6; Baig et al., 2014; Grant & Carter, 2011). The formula for imperviousness was modified from the normalized difference vegetation index as an index for urbanization. Landsat layers were provided by the United States Geological Survey (USGS) and represent reflectance imagery from late May of 2014–2016.

TOA Reflectance is given by:

\begin{eqnarray*}\rho {\lambda }^{{^{\prime}}}={M}_{\rho }{Q}_{\mathrm{cal}}+{A}_{\rho } \end{eqnarray*}

in which *ρλ*′ is Landsat band calculated for surface reflectance, *M*_*ρ*_ reflectance multi-band x (from metadata), *A*_*ρ*_ reflectance add band x (from metadata), and *Q*_cal_ original Landsat band.

TOA Corrected Reflectance is calculated as:

\begin{eqnarray*}p\lambda =\rho {\lambda }^{{^{\prime}}}/\sin \nolimits \theta \mathrm{SE} \end{eqnarray*}

where ρ*λ* corresponds to reflectance band corrected for sun elevation, and θSE sun elevation (from metadata).

Imperviousness is measured by:

\begin{eqnarray*}\mathrm{Imperviousness} \left( \mathrm{index} \right) =1- \frac{\mathrm{Band} 5-\mathrm{Band~ 4}}{\mathrm{Band~ 5}+\mathrm{Band~ 4}} . \end{eqnarray*}

The greenness, wetness, and imperviousness values were then extracted to survey points to use in linear regression models.

### Statistical analysis

A non-parametric Kruskall–Wallis one-way ANOVA test was used to compare species composition across sampling localities.

Multiple linear regression was carried out to determine significant factors for resource-weighted densities of two cicada species. The dependent variable was either area- or tree-weighted density of each species. Independent variables consisted of sampling period, minimum temperature from June to August 2014, age of residential complex, wetness, greenness, and imperviousness. Independence of residuals was examined by a Durbin-Watson test. A Durbin-Watson statistic ranging from 0 to 4 indicates no correlation between residuals. Multicollinearity was inspected by correlation coefficient and variance inflation factor (VIF). None of the correlation coefficients were greater than 0.7 (Supplemental Information 7), and all VIF values were less than 3, which confirmed no multicollinearity. Normality of the residuals was assessed visually by Q–Q plot of studentized residuals. Resource-weighed densities of both species required transformation to meet normal distribution assumption of residuals: for *C. atrata*, area-weighted density: log(*x* + 1), tree-weighted density: log(1, 000*x* + 1); for *H. fuscata*, area-weighted density: log(*x* + 1), tree-weighted density: log(10, 000*x* + 1), in which *x*  is the density of each species. Homogeneity of variance of residuals was examined using diagnostic plots of predicted values versus standardized residuals. Significant outliers were investigated using Leverage values and Cook’s distance. All Leverage values and Cook’s distances were less than 0.3, which suggested an absence of significant outliers. Overall, all assumptions of linear regression models were met. All statistical analyses were performed in SPSS 22 (IBM Corp; Armonk, NY, USA).

## Results

**Figure 2:**
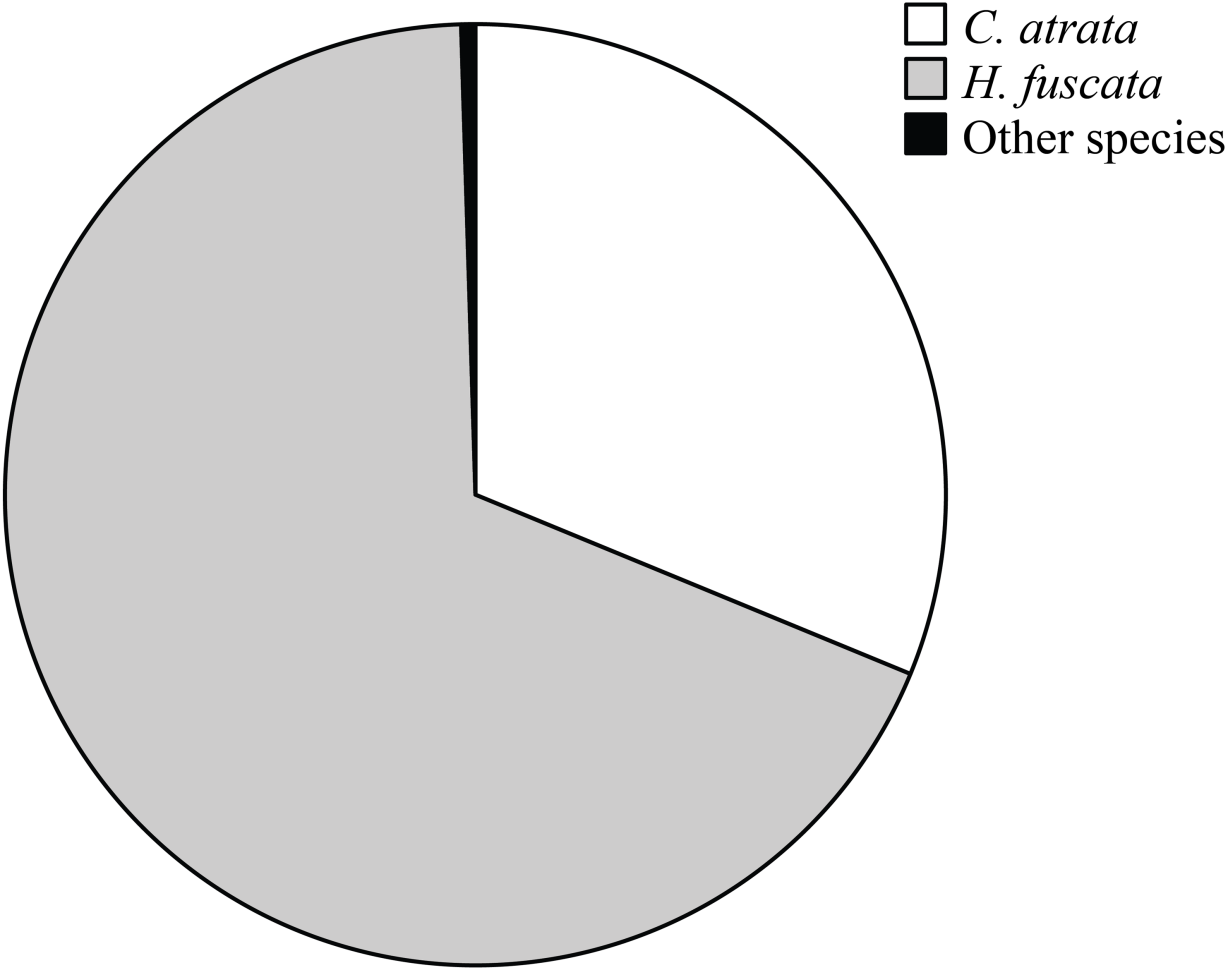
Pie chart showing cicada species composition in all localities, in which *C. atrata* composed 30%, *H. fuscata* 66%, and other species nearly 1%.

Overall, *C. atrata* and *H. fuscata* constituted most of cicada species in all localities, in which *C. atrata* comprised approximately 30%, *H. fuscata* 66%, and other species 1% (Fig. 2). Beside *C. atrata* and *H. fuscata*, exuviae of *Graptopsaltria nigrofuscata*, *Meimuna opalifera*, *Meimuna mongolica*, and *Platypleura kaemfperi* were also collected. Non-parametric tests yielded non-significant difference of species composition across localities (Kruskal–Wallis test; for *C. atrata χ*^2^ (11, *N* = 36) = 16.21, *P* = 0.133, for *H. fuscata χ*^2^(11, *N* = 36) = 11.53, *P* = 0.400, for other species *χ*^2^(11, *N* = 36) = 9.97, *P* = 0.533). Such results justified our sampling method, as species composition was not a contributing factor for the variance of cicada densities.

### Resource-weighted densities of *C. atrata*

Area-weighted density was 119.71 exuviae/km^2^/day, and tree-weighted density was 3.1 × 10^−3^ exuviae/tree/day in the first sampling period. The resource-weighted densities in the second sampling was triple those in the first sampling. Overall, densities of *C. atrata* increased with the increase in minimum temperature.

**Figure 3:**
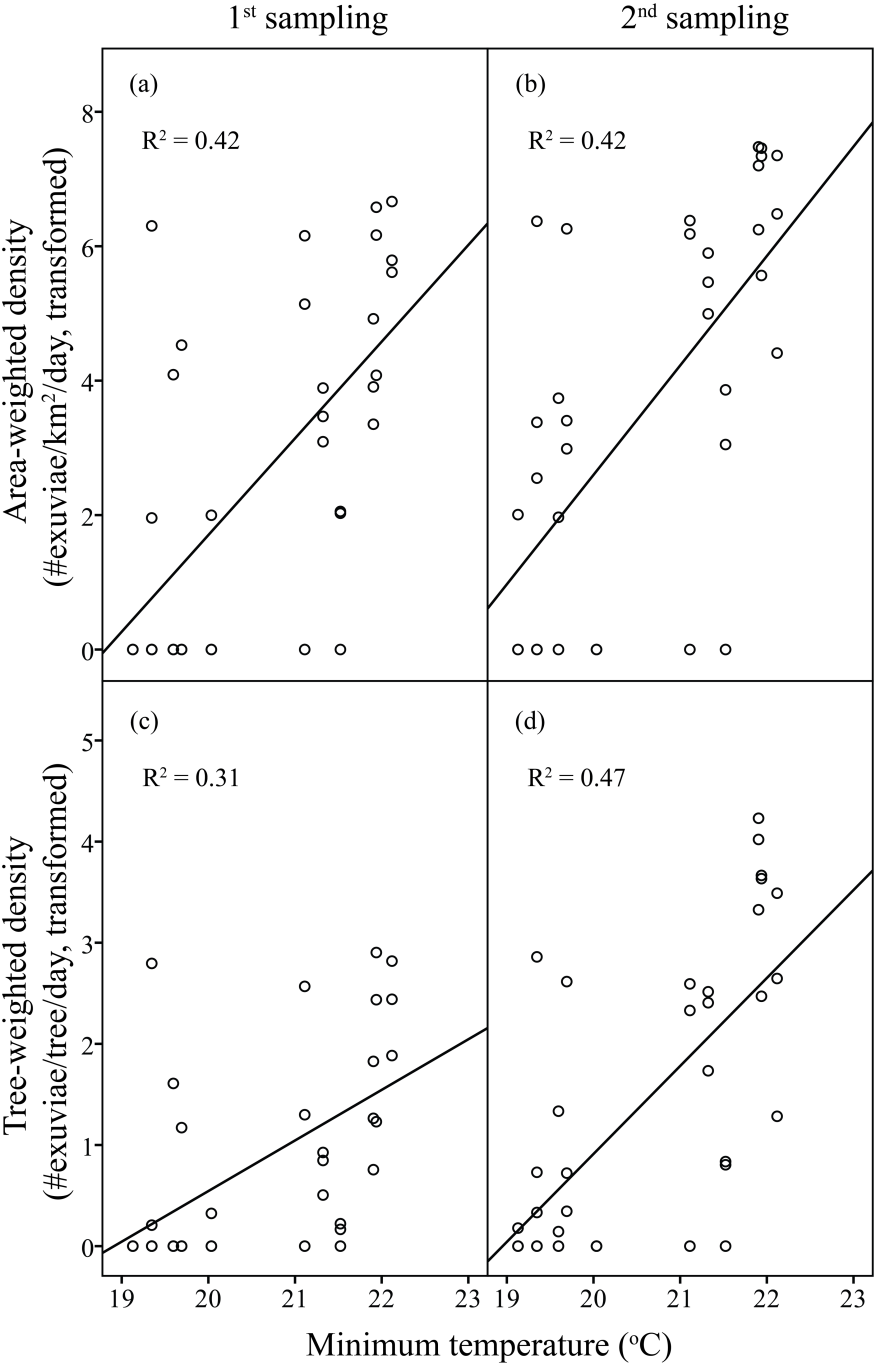
Linear regression of temperature on resource-weighted densities of *C. atrata* in two sampling periods. (A) and (B) are area-weighted densities, (C) and (D) are tree-weighted densities in the first and second sampling periods, respectively. Area-weighted density was log(*x* + 1) transformed, and tree-weighted density was log(1000x + 1) transformed. All regression models are statistically significant (*P* < 0.001). For area-weighted density, (A) *y* = 1.44x − 27.07, (B) *y* = 1.63x − 29.96; for tree-weighted density, (C) *y* = 0.50x − 9.46, (D) *y* = 0.87x − 16.48.

Linear regression models significantly predicted resource-weighted densities of *C. atrata* (area-weighted density *F*_6,65_ = 9.76, *P* < 0.001, adjusted *R*^2^ = 0.42, tree-weighted density *F*_6,65_ = 9.19, *P* < 0.001, adjusted *R*^2^ = 0.41). Sampling period and minimum temperature were significantly positively correlated to both area- and tree-weighted densities of *C. atrata* (*P* < 0.05, Table 2, Fig. 3), whereas age of residential complex and other environmental factors were not significant (*P* > 0.05, Table 2).

**Table 2:** Multiple regression analyses to test for factors critical to the abundance of *C. atrata*. Area-weighted density was log(*x* + 1) transformed, tree-weighted density was log(1,000*x* + 1) transformed. Significant results are in bold.

Dependent variable	Independent variable	${\mathbi{R}}_{\mathbi{adj}}^{2}$	*F*	Durbin-Watson	*B*	SE	*t*	*P*	VIF
Area-weighted density		0.42	9.76	1.56				**<0.001**	
	(Constant)				−29.80	5.09	−5.86	**<0.001**	
	Sampling period				1.01	0.48	2.09	**0.041**	1
	Minimum temperature				1.41	0.24	5.94	**<0.001**	1.20
	Age of residential complex				0.05	0.03	1.59	1.117	1.52
	Wetness				8.6	10.81	0.79	0.429	1.85
	Greenness				−3.76	7.39	−0.51	0.613	2.40
	Imperviousness				2.38	2.73	0.87	0.387	1.87
Tree-weighted density		0.41	9.19	1.51				**<0.001**	
	(Constant)				−13.62	2.42	−5.61	**<0.001**	
	Sampling period				0.58	0.23	2.54	**0.013**	1
	Minimum temperature				0.61	0.11	5.43	**<0.001**	1.20
	Age of residential complex				0.02	0.01	1.63	0.107	1.52
	Wetness				6.35	5.16	1.23	0.222	1.85
	Greenness				−1.72	3.52	−0.49	0.626	2.40
	Imperviousness				1.36	1.30	1.05	0.299	1.87

**Notes.**

${R}_{adj}^{2}$ adjusted coefficient of determination *B* unstandardized regression coefficient SE standardized error VIF variance inflation factor

### Resource-weighted densities of *H. fuscata*

In the first sampling, resource-weighted densities were respectively 132.18 exuviae/km^2^ /day for area- and 4.72 × 10^−3^ exuviae/tree/day for tree-weighted density. Compared to those, the resource-weighted densities in the second sampling were nine times higher. Analogous to *C. atrata*, the overall pattern represents a parallel increase between *H. fuscata* densities and elevated minimum temperature.

**Figure 4:**
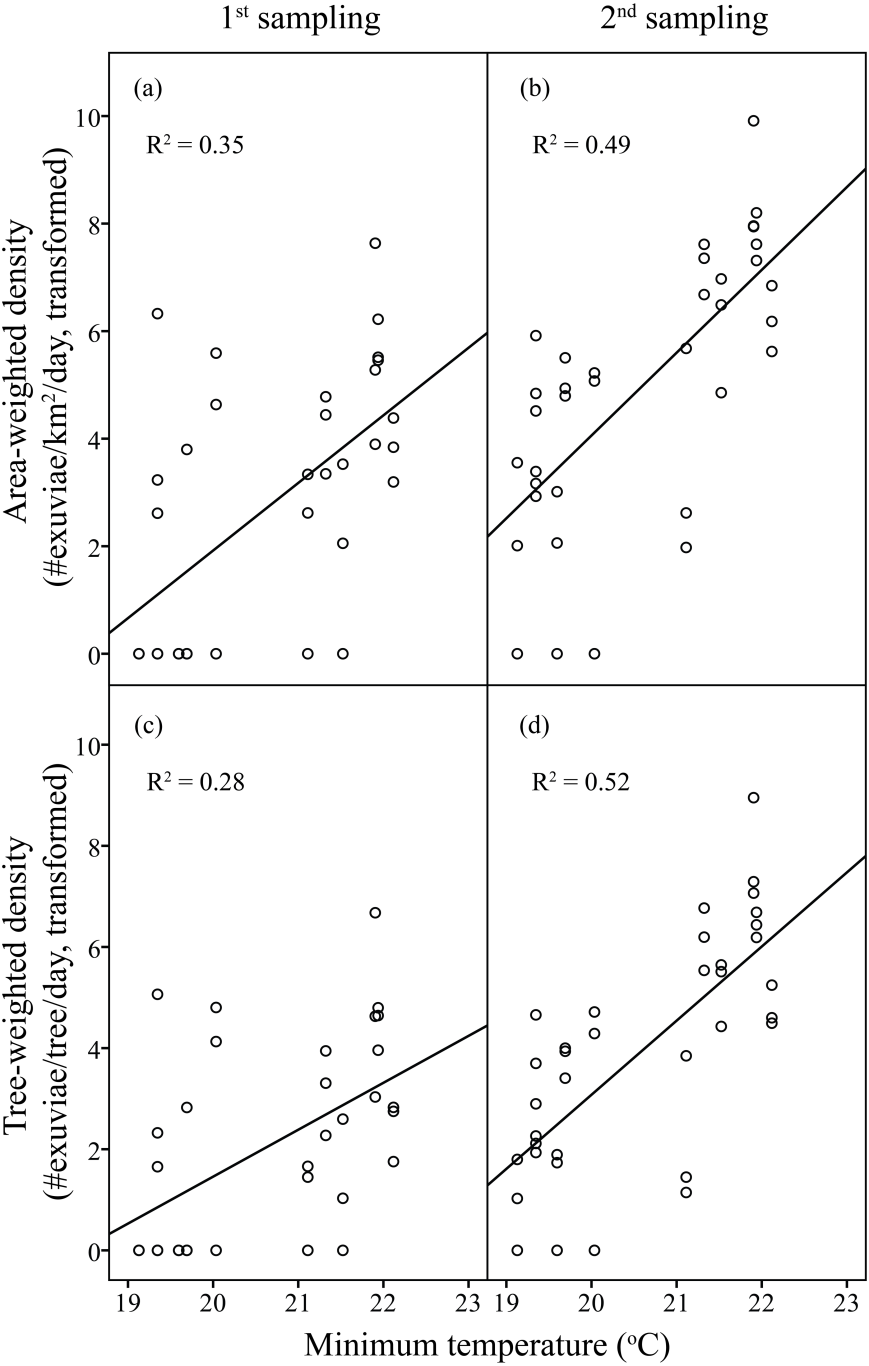
Linear regression of temperature on resource-weighted densities of *H. fuscata* in two sampling periods. (A) and (B) are area-weighted densities, (C) and (D) are tree-weighted densities in the first and second sampling periods, respectively. Area-weighted density was log(*x* + 1) transformed, and tree-weighted density was log(10000x + 1) transformed. All regression models are statistically significant (*P* < 0.001). For area-weighted density, (A) *y* = 1.23x − 23.22, (B) *y* = 1.54x − 26.74; for tree-weighted density, (C) 0.93x − 17.10, (D) *y* = 1.46x − 26.24.

**Figure 5:**
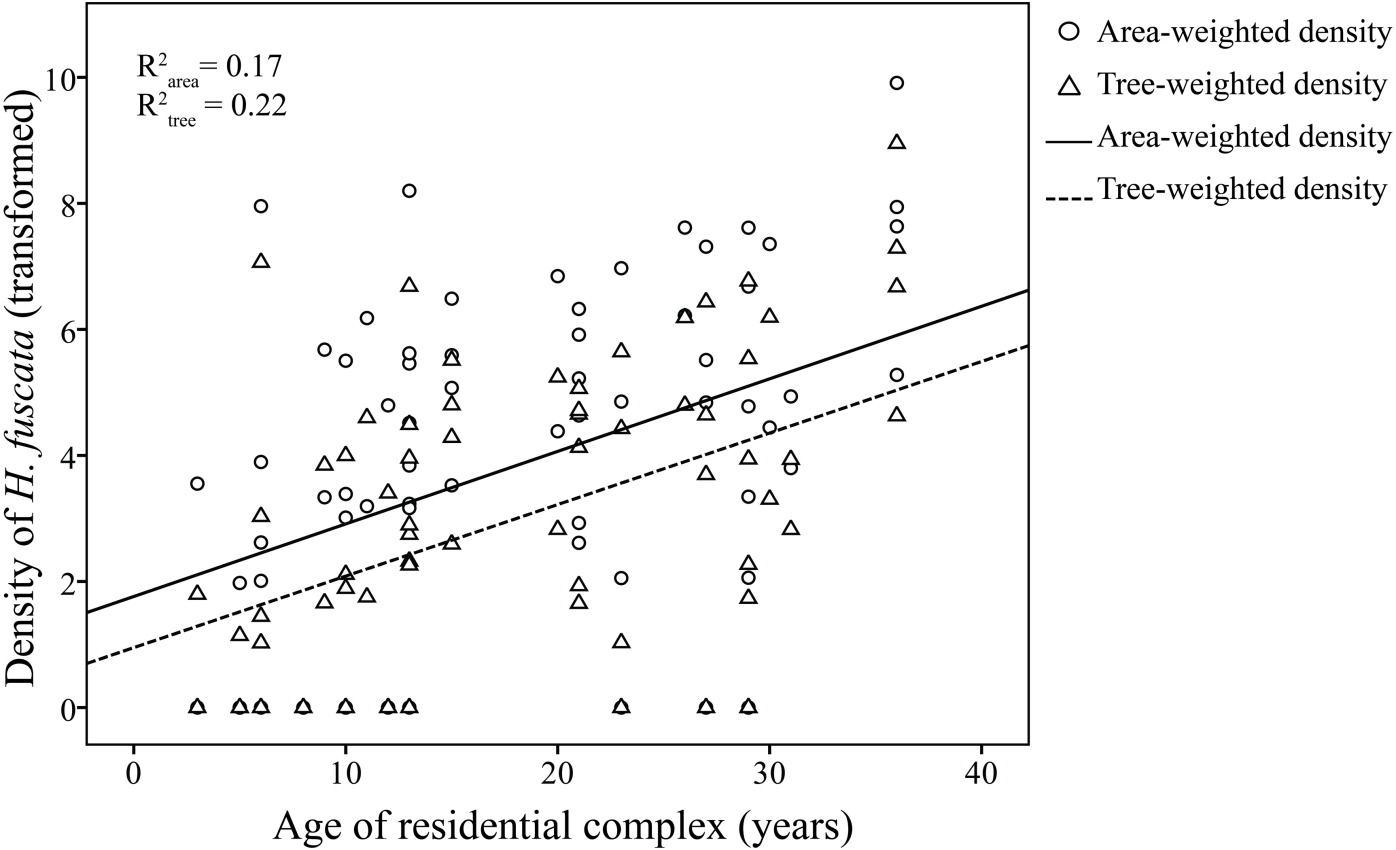
Linear regression of age of residential complex on resource-weighted densities of *H. fuscata*. Area-weighted density was log(*x* + 1) transformed, and tree-weighted density was log(10000x + 1) transformed. Two regression models are statistically significant (*P* < 0.001). For area-weighted density, *y* = 0.14x + 1.4; for tree-weighted density, *y* = 0.13x + 0.67.

Linear regression models were significant for *H. fuscata* densities (area-weighted density *F*_6,65_ = 18.93, *P* < 0.001, adjusted *R*^2^ = 0.60, tree-weighted density *F*_6,65_ = 19.97, *P* < 0.001, adjusted *R*^2^ = 0.62). Again, sampling period and minimum temperature were significant variables for resource-weighted densities (*P* < 0.001, Table 3, Fig. 4). In addition, age of residential complex did not exhibit a significant correlation to *C. atrata* densities, but to *H. fuscata* densities (*P* < 0.001, Table 3, Fig. 5). Besides minimum temperature, no other environmental factors were found significant (*P* > 0.05, Table 3).

**Table 3:** Multiple regression analyses to test for factors critical to the abundance of *H. fuscata*. Area-weighted density was log(*x* + 1) transformed, tree-weighted density was log(10,000*x* + 1) transformed. Significant results are in bold.

Dependent variable	Independent variable	${\mathbi{R}}_{\mathbi{adj}}^{2}$	*F*	Durbin-Watson	*B*	SE	*t*	*P*	VIF
Area-weighted density		0.60	18.93	1.22				**<0.001**	
	(Constant)				−25.47	4.22	−6.03	**<0.001**	
	Sampling period				2.31	0.40	5.76	**<0.001**	1
	Mean temperature				1.06	0.2	5.40	**<0.001**	1.20
	Age of residential complex				0.11	0.03	4.26	**<0.001**	1.52
	Wetness				16.47	8.97	1.83	0.071	1.85
	Greenness				2.51	6.13	0.41	0.684	2.40
	Imperviousness				3.22	2.27	1.42	0.161	1.87
Tree-weighted density		0.62	19.97	1.1				**<0.001**	
	(Constant)				−21.78	3.60	−6.05	**<0.001**	
	Sampling period				1.94	0.34	5.67	**<0.001**	1
	Mean temperature				0.88	0.17	5.26	**<0.001**	1.20
	Age of residential complex				0.11	0.02	5.01	**<0.001**	1.52
	Wetness				13.81	7.65	1.80	0.076	1.85
	Greenness				0.92	5.23	0.18	0.861	2.40
	Imperviousness				2.81	1.94	1.45	0.152	1.87

**Notes.**

${R}_{adj}^{2}$ adjusted coefficient of determination *B* unstandardized regression coefficient SE standardized error VIF variance inflation factor

## Discussion

In sum, our results demonstrate that high temperature is related to high cicada densities in metropolitan Seoul. A significant positive correlation is found between cicada densities and temperature, but not to any other environmental variables contributing to urbanization. The dispersal ability of cicadas is poor (Karban, 1981) and they have a prolonged development time as nymphs underground, both of which lead them to experience and respond measurably to fluctuations in urban temperature. Moreover, cicadas’ neuromuscular apparatus (Fonseca & Revez, 2002), diapause development (Moriyama & Numata, 2008), and species distribution (Toolson, 1998) are highly dependent on temperature. Hence, this result further strengthens the hypothesis attributing UHI effect to the cicada outbreak in metropolitan Seoul.

The sampling period plays a critical role in elucidating the pattern of emergence in cicadas. In metropolitan Seoul, the onset of cicada emergence begins in the middle of July, which coincides with our first sampling, and mass emergence occurs in the beginning of August, the time of our second sampling. This phenological pattern is well reflected in our study, and also displayed in cicadas of Japan (Sato & Sato, 2015). In their research, Sato and his colleague (2015) also found an earlier emergence of males than females, which might facilitate males to gain mating opportunities for females. However, such intersexual phenological difference was not exhibited in our study.

There is mounting evidence for a positive correlation between UHI intensity and abundance of other herbivorous insects (Dale & Frank, 2014a; Meineke et al., 2013; Youngsteadt et al., 2014), which corroborates UHI as an important environmental habitat condition for the population dynamics of such organisms. Although the underlying mechanistic link between UHI and the observed population explosion of the studied cicadas remains to be determined, there are several direct and indirect consequences of UHI that potentially foster the prevalence of cicadas in warm urban cores. Of these, a highfecundity of females and a reduced mortality rate of nymphs are most likely initiated by urban warming. Within non-stressful rearing conditions, elevated temperatures increase insect metabolic activity, thus elevating the reproductive success of numerous insects, such as the leafminer *Liriomyza trifolii* (Leibee, 1984), cotton aphid *Aphis gossypii* (Kersting, Satar & Uygun, 1999), lace bug *Corythucha ciliata* (Ju, Wang & Li, 2011), scale insect *Melanaspis tenebricosa* (Dale & Frank, 2014b), and silverleaf whitefly *Bemisia tabaci* (Butler Jr, Henneberry & Clayton, 1983). Reduction in mortality rate of instars of various insects in conjunction with improved rearing conditions is also observed in other insect species, such as the aphid *Myzus persicae* (Bale, Harrington & Clough, 1988), mosquitoes *Culex quinquefasciatus* and *Aedes aegypti* (Rueda et al., 1990), pine processionary moth *Thaumetopoea pityocampa* (Battisti et al., 2005), and cotton aphids (Kersting, Satar & Uygun, 1999). Additionally, higher rearing temperatures result in bigger body sizes of some insects (Atkinson, 1994), which could promote survival, mating success, and fecundity (Kingsolver & Huey, 2008; Honěk, 1993), and eventually result in higher population density. We also observed a significantly bigger thorax width of *H. fuscata* females in the high UHI group compared to those in the low UHI group, whereas no difference was shown among *C. atrata* individuals (HQ Nguyen, 2016, unpublished data). Thus, the relatively large body size of *H. fuscata* inhabiting high UHI localities in metropolitan Seoul may lead to high fecundity, and ultimately to higher population density.

Herbivorous insect outbreak in urban habitats may also be exacerbated by the deterioration in host plant quality. Trees planted in urban areas tend to face upwards because of dehydration due to soil compaction, confined space planting and impervious surfaces (Raupp, Shrewsbury & Herms, 2010). Such induced water stress increases the concentration of soluble nitrogen in phloem sap, which is generally restricted under normal water conditions, and promotes insect outbreak (Huberty & Denno, 2004; White, 1984). Nevertheless, the impact of host quality on the performance of herbivorous insects also depends on types of feeding guilds (Huberty & Denno, 2004; Larsson, 1989). Both positive and negative responses in fecundity and population growth of sucking insects to water-stress plants have been reported (Koricheva, Larsson & Haukioja, 1998), which makes it difficult to generalize such effects on sucking insect performance. Separate research to investigate exactly how these two cicada species actually respond to water-stress conditions of their host plants will be helpful to disentangle such a causal relationship.

We also consider the adaptive evolution of individual species to local habitat conditions as a possible factor for the abundance of two cicada species in urban areas. Evidence of such a phenomenon is reported in the scale insect *Parthenolecanium quercifex* (Meineke et al., 2013). Both source and common-garden populations of *P. quercifex* indicated local adaptation of this species to warming. In the source populations, densities increased measurably under hot conditions. In common-garden populations, individuals collected from trees in hot urban areas were twice as abundant as individuals from trees in cool urban areas, regardless of rearing condition; whereas those from cool trees did not become more abundant when reared in hot conditions. If adaptation to local habitat at a small spatial scale leads to the observed abundance patterns of two cicada species, intraspecific differentiation of organisms in both phenotypic plasticity and genetic basis will be further manifested (Rank, Dahlhoff & Fenster, 2002).

## Conclusion

Overall, we have found a positive relationship between the abundance of two cicada species and UHI intensities in urban areas. More work is necessary to elucidate the influence of UHI on the fecundity of females and the survivorship of nymphs, to examine the interaction between body size and reproductive success under the effect of UHI, and to determine the actual impact of plant stress on the performance of cicadas inhabiting areas of heterogeneous UHI indices. Studies on effects of genetic basis and habitat of origin should also be conducted to confirm UHI as the main factor causing high density of cicadas in urban areas.

##  Supplemental Information

10.7717/peerj.4238/supp-1 Supplemental Information 1 Independent-samples *t*-test

10.7717/peerj.4238/supp-2 Supplemental Information 2 Kruskal–Wallis test

10.7717/peerj.4238/supp-3 Supplemental Information 3 Multiple linear regression

10.7717/peerj.4238/supp-4 Supplemental Information 4 Independent-samples *t*-tests for comparison of minimum and maximum temperatures between urban and suburban weather stations in summer 2014

10.7717/peerj.4238/supp-5 Supplemental Information 5 Independent-samples *t*-tests for comparison between number of males and females of each species collected in each sampling period

10.7717/peerj.4238/supp-6 Supplemental Information 6 Table of correlation coefficients for greenness and wetness tasseled cap transformations

10.7717/peerj.4238/supp-7 Supplemental Information 7 Table of correlation coefficients of all independent variables

## Additional Information and Declarations

### Data Availability

The following information was supplied regarding data availability: The raw data has been provided in the Supplemental Files.
