# Supplementary material for: Urban heat island effect on cicada densities in metropolitan Seoul
Source: PeerJ. 2018 Jan 12;6:e4238. doi: 10.7717/peerj.4238 (PMC5768176; doi:10.7717/peerj.4238)
Supplement: Supplemental Information 4 [file peerj-06-4238-s004.docx]

**Supplementary S1.** Independent-samples t-tests for comparison of minimum and maximum temperatures between urban and suburban weather stations in summer 2014. df = degree of freedom.

| Variable | Levene’s test for equality of variances | | t-tests for equality of means | | | |
| --- | --- | --- | --- | --- | --- | --- |
|  | F | *P* | t | df | *P* | Mean Difference |
| Minimum temperature | 2.18 | 0.148 | 2.28 | 36 | 0.029 | 0.86 |
| Maximum temperature | 0.27 | 0.869 | 1.72 | 36 | 0.093 | 0.45 |
