# Supplementary material for: Urban heat island effect on cicada densities in metropolitan Seoul
Source: PeerJ. 2018 Jan 12;6:e4238. doi: 10.7717/peerj.4238 (PMC5768176; doi:10.7717/peerj.4238)
Supplement: Supplemental Information 5 [file peerj-06-4238-s005.docx]

**Supplementary S2.** Independent-samples t-tests for comparison between number of males and females of each species collected in each sampling period. df = degree of freedom.

| Species | Sampling period | Levene’s test for equality of variances | | t-tests for equality of means | | | |
| --- | --- | --- | --- | --- | --- | --- | --- |
|  |  | F | *P* | t | df | *P* | Mean Difference |
| *Cryptotympana atrata* | 1^st^ | 4.05 | 0.048 | 0.99 | 59 | 0.324 | 3.61 |
|  | 2^nd^ | 0.11 | 0.738 | -0.14 | 70 | 0.888 | -1.47 |
| *Hyalessa fuscata* | 1^st^ | 1.43 | 0.236 | 0.84 | 70 | 0.403 | 5.33 |
|  | 2^nd^ | 0.51 | 0.477 | 0.53 | 70 | 0.598 | 31.61 |
