# Supplementary material for: Urban heat island effect on cicada densities in metropolitan Seoul
Source: PeerJ. 2018 Jan 12;6:e4238. doi: 10.7717/peerj.4238 (PMC5768176; doi:10.7717/peerj.4238)
Supplement: Supplemental Information 6 [file peerj-06-4238-s006.docx]

**Supplementary S3.** Table of correlation coefficients for greenness and wetness tasseled cap transformations.

|  | Band 2 | Band 3 | Band 4 | Band 5 | Band 6 | Band 7 |
| --- | --- | --- | --- | --- | --- | --- |
| Greenness | -0.2941 | -0.243 | -0.5424 | 0.7276 | 0.0713 | -0.1608 |
| Wetness | 0.1511 | 0.1973 | 0.3283 | 0.3407 | -0.7117 | -0.4559 |
