# Supplementary material for: Urban heat island effect on cicada densities in metropolitan Seoul
Source: PeerJ. 2018 Jan 12;6:e4238. doi: 10.7717/peerj.4238 (PMC5768176; doi:10.7717/peerj.4238)
Supplement: Supplemental Information 7 [file peerj-06-4238-s007.docx]

**Supplementary S4.** Table of correlation coefficients of all independent variables. Significant results are in bold.

|  | | Sampling period | Minimum temperature | Age of building | Wetness | Greenness | Impervious surface |
| --- | --- | --- | --- | --- | --- | --- | --- |
| Sampling period | Pearson Correlation | 1 | < 0.001 | < 0.001 | < 0.001 | < 0.001 | < 0.001 |
|  | *P* |  | 1 | 1 | 1 | 1 | 1 |
|  | N | 72 | 72 | 72 | 72 | 72 | 72 |
| Minimum temperature | Pearson Correlation | < 0.001 | 1 | **0.26** | 0.18 | 0.14 | -0.02 |
|  | *P* | 1 |  | **0.026** | 0.137 | 0.238 | 0.855 |
|  | N | 72 | 72 | **72** | 72 | 72 | 72 |
| Age of building | Pearson Correlation | < 0.001 | **0.26** | 1 | **-0.38** | 0.1 | -0.21 |
|  | *P* | 1 | **0.026** |  | **0.001** | 0.421 | 0.083 |
|  | N | 72 | **72** | 72 | **72** | 72 | 72 |
| Wetness | Pearson Correlation | < 0.001 | 0.18 | **-0.38** | 1 | 0.46 | -0.13 |
|  | *P* | 1 | 0.137 | **0.001** |  | **< 0.001** | 0.267 |
|  | N | 72 | 72 | **72** | 72 | 72 | 72 |
| Greenness | Pearson Correlation | < 0.001 | 0.14 | 0.1 | 0.46 | 1 | -0.65 |
|  | *P* | 1 | 0.238 | 0.421 | **< 0.001** |  | **< 0.001** |
|  | N | 72 | 72 | 72 | 72 | 72 | 72 |
| Impervious surface | Pearson Correlation | < 0.001 | -0.02 | -0.21 | -0.13 | -0.65 | 1 |
|  | *P* | 1 | 0.855 | 0.083 | 0.267 | **< 0.001** |  |
|  | N | 72 | 72 | 72 | 72 | 72 | 72 |
